# Preventable Factors in Fatal and Near-Fatal Asthma: Lessons From the British Thoracic Society (BTS) and the National Review of Asthma Deaths (NRAD)

**DOI:** 10.7759/cureus.114017

**Published:** 2026-08-05

**Authors:** Muhammad Meer, Mahnoor Mumtaz, Syed Zuhair, Nafiu Mukhtar

**Affiliations:** 1 Acute Medicine, Northampton General Hospital, Northampton, GBR; 2 Internal Medicine, King's College Hospital NHS Foundation Trust, London, GBR; 3 General Medicine, Northampton General Hospital, Northampton, GBR

**Keywords:** asthma, asthma deaths, bts/sign guidelines, near-fatal asthma, nrad

## Abstract

Asthma is one of the most widespread chronic respiratory illnesses that is still a leading cause of high morbidity and mortality even in the context of effective therapies and evidence-based clinical practice. The British Thoracic Society (BTS)/Scottish Intercollegiate Guidelines Network (SIGN) provides a structured guideline on the management of asthma, and the National Review of Asthma Deaths (NRAD) has outlined the most significant gaps in routine asthma care that lead to preventable deaths. The purpose of the review is to consider the preventable aspects related to fatal and near-fatal asthma and to comment on the major learning points from the BTS/SIGN asthma guidelines and the results of the NRAD. A narrative database literature review was carried out using PubMed, Scopus, and Google Scholar to obtain relevant peer-reviewed literature published since 2005 till December 2025. Search words were fatal asthma, near-fatal asthma, asthma mortality, preventable factors, NRAD, and the BTS asthma guideline. Articles on asthma mortality, severe exacerbations, and asthma management using the guidelines were selected. The literature recognizes various preventable causes of fatal and near-fatal asthma; among them are poor adherence to inhaled corticosteroid treatment, overuse of short-acting β2-agonists (SABA), the presence of poor follow-up following an asthma attack, absence of individualized asthma action plans, and failure to recognize worsening symptoms. Findings in the NRAD research also indicated the shortcomings of standard check-ups of asthma, minimal use of controller drugs, and the inability to recognize at-risk patients as the key contributors to asthma death. It is possible to prevent many asthma deaths by increasing observance of guidelines, early recognition of high-risk patients, and providing them with more education and self-management support. Strengthening the implementation of the BTS/SIGN guidelines and addressing gaps in NRAD insights could be key to improving the quality of asthma care and preventing asthma-related deaths.

## Introduction and background

Global burden of asthma

Asthma is a chronic inflammatory airway disease characterized by variable and reversible airway obstruction, airway hyperresponsiveness, and recurrent respiratory symptoms, including wheezing, dyspnea, chest tightness, and cough [1,2]. It is one of the most prevalent chronic respiratory diseases worldwide and imposes a significant burden on global health systems [3]. According to epidemiological data, asthma affects hundreds of millions of people worldwide, and it remains a leading cause of morbidity in all age groups [4,5]. A major economic burden on healthcare systems is the disease, since individuals consult outpatient clinics regularly, visit the emergency department repeatedly with numerous emergency hospitalizations, and face the burden of drug treatment over a long period [6,7].

Asthma prevalence continues to rise globally, and despite effective therapies, the disease causes hundreds of thousands of deaths annually [3,8]. In severe cases of asthma attacks, respiratory failure can develop very fast before it is noticed, which makes it highly important to address the situation in the first place and find the right long-term management strategies [7]. Despite the common belief that asthma is a disease that can be treated and managed, the persistence of preventable deaths indicates that gaps remain in patient education, adherence to guidelines, and healthcare delivery systems [1,5].

Asthma mortality and severe exacerbations

The most frequent condition linked with asthma death is a severe acute exacerbation resulting in progressive airflow obstruction, respiratory failure, and eventual death unless the disease is treated in time [9]. The attacks of severe asthma are readily worsened by severe bronchoconstriction, airway inflammation, and mucus plugging, which severely diminish both ventilation and gas exchange [10,11]. Fatal asthma, in most instances, is a case where acute exacerbations lead to critical respiratory failure of respiratory hypoxia/hypercapnia, which cannot be reversed through medical attention [12].

Fatal asthma is a type of asthma exacerbation, which in turn leads to death due to respiratory failure or infection-related complications related to severe airway obstruction [13,14]. These incidents are usually marked by a dramatic clinical decline, extreme pulmonary blockage, and insufficient reaction to a bronchodilator inhaler and other emergent care [9]. Research has revealed that a significant number of fatal cases of asthma happen at home or prior to sufficient emergency treatment being administered, which further leads to deaths [14,15].

Near-fatal asthma is a life-threatening form of asthma exacerbation during which patients experience severe respiratory compromise that necessitates an aggressive form of medical intervention to avoid death [11,16]. In clinical practice, near-fatal asthma is usually characterized by respiratory arrest, extreme hypercapnia, or acute respiratory failure, which necessitates intubation and mechanical ventilation [13,17]. Severe bronchospasm and inability to exchange gases often lead to the admission of many patients with near-fatal asthma to intensive care units and sophisticated respiratory assistance [10,18].

Notably, near-fatal asthma events are viewed as effective predictors of asthma mortality in the future since such incidences are indicators of severe underlying disease and inability to control airway inflammation [14,19]. It has been indicated that a large percentage of patients who succumb to asthma have undergone severe exacerbations in the past that necessitated hospitalization, an emergency, or mechanical ventilation [13,20]. Thus, the detection of patients who have already had a near-fatal attack or experienced recurrent severe exacerbation is essential to the prevention of asthma-related mortality and enhanced management of the disease over the long term [10,12].

Preventable nature of many asthma deaths

There is a significant amount of evidence suggesting that several deaths related to asthma can be avoided and are often linked to the modification of patient care and healthcare concerns [21,22]. The examination of asthma mortality has continuously shown that lapses in primary care, late identification of progressive symptoms, and insufficient disease management over the long run are considerable contributors to death [23,24]. The National Review of Asthma Deaths (NRAD) in the United Kingdom found that major potentially avoidable factors were identified in 67% of confirmed asthma deaths, highlighting significant gaps in clinical practice and patients' asthma self-management [21,25]. 

Poor adherence to prescribed controller medications, especially inhaled corticosteroids (ICS), which are critical for maintaining airway inflammation and preventing severe exacerbations, has to be considered among the most prevalent preventable factors [26,27]. Overuse of short-acting β2-agonist (SABA) reliever inhalers has been closely linked to the development of both asthma attacks and death, as people tend to use them too frequently and overlook the progressive airway inflammation without treating the disease process [25,28]. Moreover, the lack of correct clinical follow-up and insufficient asthma control monitoring may lead to a repetition of a plan change and subsequently promote severe attacks and negative outcomes [23,24]. Late attendance of medical care with deteriorating symptoms and lack of individualized asthma action plans also add to preventable morbidity and mortality by reducing the possibility of early intervention in acute attacks [21,26].

Notably, these factors can be addressed at several levels of asthma treatment, including patient factors, factors related to healthcare professionals, and factors related to the healthcare system [21,22]. Healthcare provider-related factors can include inadequate assessment of asthma control, inappropriate prescribing practices, and insufficient written asthma action plans and education on inhaler technique [23,27]. These results, in total, demonstrate numerous asthma deaths attributable to nonadherence to routine asthma care, underscoring the need to enhance adherence to a guideline-based approach to disease treatment and improve patient education [24,27].

Importance of clinical guidelines in asthma management

Clinical practice guidelines are essential for improving the diagnosis, treatment, and long-term management of asthma because they provide evidence-based, standardized guidance for healthcare professionals [29,30]. They are developed through the systematic evaluation of the scientific literature and are intended to optimize patient outcomes through consistent and effective clinical decision-making [31,32]. Adherence to evidence-based asthma recommendations has been associated with better disease control, fewer exacerbations, and reduced healthcare utilization [29,33].

A range of international and national guidelines support asthma management, with the Global Initiative for Asthma (GINA) and UK national guidance being among the most widely used [30,34]. GINA remains an internationally recognized framework for asthma diagnosis, prevention, and treatment, with particular emphasis on personalized care and longitudinal assessment of disease control [30,35]. Historically, the British Thoracic Society (BTS)/Scottish Intercollegiate Guidelines Network (SIGN) guideline has been central to asthma care in the United Kingdom [36,37].

UK national asthma guidance places particular emphasis on risk assessment to identify patients at increased risk of severe exacerbations or asthma-related mortality. One of its key principles is that ICS form the foundation of asthma treatment by reducing airway inflammation and preventing attacks [37,38]. In addition, the guideline supports the use of personalized written asthma action plans to improve self-management and facilitate early recognition of worsening symptoms [39]. Routine monitoring and regular clinical review are also emphasized to assess treatment response, evaluate adherence, and adjust therapy when needed [36,37]. More recently, these principles have been carried forward in the joint BTS/National Institute for Health and Clinical Excellence (NICE)/SIGN 2024 asthma guideline [40]. Taken together, these guideline-based measures provide a structured framework for improving asthma care and reducing severe exacerbations and preventable deaths [30,33].

NRAD

The big study was the NRAD, which examined the situation in the United Kingdom to investigate factors that might have led to asthma-related deaths. The review was ordered to assess clinical care before the occurrence of lethal asthma attacks and whether gaps in healthcare services or disease control contributed to the death of asthma [22,41]. One of the purposes of NRAD was to identify potentially preventable factors associated with asthma deaths and to provide recommendations to enhance asthma care at the healthcare system level [21,24].

The NRAD study in the United Kingdom received 276 cases for confidential enquiry, of which 195 were confirmed asthma deaths and analyzed, along with comprehensive medical, treatment, and health contact histories before death [21,41]. The results indicated that the percentage of asthma deaths could be reduced, suggesting severe deficits in asthma control and management [22]. The review has identified several modifiable factors that contribute to mortality in primary and secondary care settings and highlighted the need to enhance clinical practice and patient management [21,24].

One of the issues, most noticeable in the NRAD report, was the lack of frequent reviews of asthma cases, which restricted healthcare providers' ability to assess disease control and make necessary changes [21,41]. Overuse of SABA inhalers, which is linked to ineffective asthma management and dangerous exacerbation, was also mentioned in the review. Conversely, underutilization of ICS as one of the main preventive therapies for asthma was also common in patients who perished due to asthma [22,41].

Also, the NRAD report stated that many healthcare providers identified the problem of missing patients at risk of asthma-related mortality, thereby missing an opportunity to intervene early and monitor patients more closely [21,24]. Together, these results demonstrated critical gaps in applying the BTS/SIGN guideline recommendations and that better compliance with evidence-based asthma management could help prevent deaths associated with asthma [21].

Relationship between BTS guidelines and NRAD findings

The BTS/SIGN guidelines, and more recently the BTS/NICE/SIGN 2024 guideline, provide evidence-based guidance on the diagnosis, monitoring, and treatment of asthma across healthcare settings [36,37,40]. These recommendations highlight key strategies to improve asthma outcomes, including regular assessment of disease control, appropriate use of ICS, identification of high-risk patients, and provision of individualized asthma action plans [34,37]. The updated 2024 guidance is particularly relevant because it allows the lessons of NRAD to be reconsidered in the context of current clinical standards.

The evidence from the NRAD showed that a great number of patients who died because of asthma did not receive treatment according to the recommendations of the appropriate guidelines [21,41]. The review showed that the common failures in performing the essential aspects of asthma management, in the form of regular follow-up, proper prescription of ICS, and recognition of high-risk individuals, were typical among the fatal cases of asthma [21,22]. These results imply that the continued existence of asthma mortality does not necessarily indicate the absence of efficient guidelines, but rather reflects loopholes in their application in everyday clinical practice [24,33].

In turn, the NRAD findings confirm that compliance with the BTS/SIGN guidelines remains a crucial factor in the daily management of asthma to prevent preventable deaths [21]. Examining the relationship between BTS/SIGN guidelines and NRAD outcomes enables the identification of modifiable risk factors contributing to asthma-related deaths and pinpointing deficiencies in clinical practice that could negatively impact patient outcomes [22]. Furthermore, integrating insights from NRAD with established guideline recommendations can guide the development of targeted strategies to alleviate the asthma burden and enhance guideline adherence, thereby preventing preventable asthma fatalities [21,33].

Taken together, NRAD identifies the recurring failures associated with asthma deaths, while current UK guidance provides a framework for addressing many of these shortcomings. However, persistent implementation gaps remain, particularly in routine review, recognition of risk, prescribing behavior, and supported self-management.

Rationale for the review

Despite major advances in asthma pharmacotherapy and the availability of evidence-based guidance, preventable asthma deaths continue to occur. This indicates persistent gaps in long-term asthma monitoring, recognition of deterioration, use of preventive treatment, and implementation of guideline-based care [4,21,23,24]. A focused synthesis of these preventable factors remains important, particularly in light of NRAD and subsequent updates in asthma guidance.

The NRAD offers one of the most comprehensive analyses of asthma deaths and can be useful for revealing details of clinical practice before fatal asthma events in the real world [21]. The results of NRAD illustrate some of the clinical and system-related deficiencies that can be improved and that cause preventable deaths among asthma patients, such as improper monitoring, incorrect prescribing practices, and inadequate patient education [22]. To comprehend these results, it is necessary to identify gaps in asthma management and to explain specific interventions that can enhance patient outcomes [21,27]. Moreover, lessons from NRAD can offer valuable insights to improve asthma management not only in the United Kingdom but also in other healthcare systems worldwide [23].

Aim of the review

This review focuses on the preventable factors related to fatal and near-fatal asthma, using the NRAD as a starting point, while also considering current asthma management guidelines. In particular, this review aims to summarize knowledge on the risk factors that can be modified to reduce the risk of asthma mortality, synthesize the findings of NRAD in relation to clinical and systemic failures, and evaluate how BTS/SIGN guidelines can be used to improve the management of asthma patients and eliminate life-threatening outcomes. Moreover, the review aims to identify opportunities to improve adherence to guidelines, patient management, and, eventually, preventable asthma deaths through better clinical practice and interventions in the healthcare system.

## Review

Methods

Literature Search Strategy

A narrative review methodology was used. PubMed, Scopus, and Google Scholar were searched for relevant peer-reviewed literature published from 2005 till December 2025. Search terms included fatal asthma, near-fatal asthma, asthma mortality, preventable factors, NRAD, and BTS asthma guideline. Titles and abstracts were screened for relevance, followed by full-text review of selected articles by the authors. Articles were included if they addressed asthma mortality, near-fatal asthma, severe exacerbations, preventable care factors, NRAD findings, or guideline-based asthma management. The evidence was synthesized narratively, with emphasis on recurring clinical and system-related themes, NRAD findings, and the relationship between these findings and current guideline recommendations. As this was a narrative review, no formal risk-of-bias assessment, meta-analysis, meta-regression, or quantitative pooled synthesis was performed.

Review Methodology and Study Selection

The studies identified in the database search were filtered to determine their relevance to the objectives of this review. Articles were included where they were studies that researched fatal asthma, near-fatal asthma, asthma-related mortality, or preventable factors that lead to severe asthma outcomes. Studies reviewing NRAD findings or discussing guideline-based asthma management, particularly those referring to BTS/SIGN recommendations, were also considered eligible for inclusion.** **English-language articles published from 2005 to December 2025 were considered for inclusion.

Excluded studies included non-human experimental research, non-peer-reviewed publications, conference abstracts, editorials, and commentaries without sufficient scientific data. Articles that dedicated all their attention to pediatric asthma and had no connection with risk factors of asthma mortality were also left out. The retrieved studies had their titles and abstracts screened, and the potentially eligible articles were further screened by reviewing their full texts to ascertain their suitability for inclusion in this review.

Epidemiology of fatal and near-fatal asthma

Global Prevalence and Mortality

Asthma is a highly prevalent chronic respiratory illness in the world and a major cause of global morbidity and mortality [4,42]. Epidemiological research approximates that about 300 million people have asthma worldwide and the prevalence rates are diverse in relation to geographical locations and people [1,3]. Over the past decades, the asthma burden in the entire world has been growing because of urbanization, environmental exposures, and better awareness and diagnosis of the disease [5,43]. Although the pharmacologic treatment of the disease has been improved and evidence-based control measures have been introduced, the prevalence of asthma in healthcare and hospitalization, as well as emergency visits, remains very high globally [44,45].

The mortality associated with asthma is a pressing issue in the area of social health, especially where access to medical services is low and the ways of managing the disease are inefficient [5,46]. According to Global Burden of Disease (GBD) research, asthma is a leading cause of hundreds of thousands of deaths per year, and the mortality rate in high-income as well as in low- and middle-income countries is highly inconsistent [8,45]. Asthma is a frequent cause of mortality, which is mostly due to severe exacerbations resulting in acute respiratory failure when medical intervention is not given in time and in the appropriate manner [42,47]. It is important to mention that a large part of such deaths can be prevented through the increased control of the disease, correct usage of controller medications, and effective implementation of asthma management according to the guidelines [5,21].

High-Risk Populations

There is a group of biological, environmental, and healthcare-related factors that predetermine the occurrence of severe attacks of asthma and asthma-related mortality [4,43]. Asthmatic patients with severe or poorly controlled asthma, especially those with frequent attacks or those who have previously been hospitalized, are one of the groups at risk of fatal and near-fatal asthma attacks [45,47]. A history of previous asthma attacks that necessitate mechanical ventilation or intensive care hospitalization is also associated with an increased risk of future fatal complications [13,42].

Socioeconomic conditions are also significant determinants of the risk of asthma mortality, since people with low income or belonging to an underserved group are commonly exposed to a lack of healthcare access, insufficient disease management, and insufficient adherence to preventive treatment [44,46]. Moreover, smoking, occupational exposures, air pollution, and an inability to take medication properly are other behavioral and environmental factors that increase the risk of severe outcomes of asthma [8,43]. There are also age-related differences, and older adults and young individuals with poorly controlled asthma have proven to be more vulnerable to severe exacerbation and mortality [4,45]. It is necessary to identify these high-risk populations to apply specific interventions that would decrease the cases of preventable asthma-related deaths.

Trends in Asthma Deaths

The trends in mortality due to asthma around the world have also significantly evolved over the last few decades, with the increase in the efficiency of managing asthma and prevention therapy [48,49]. Global comparisons of mortality data have also shown that the mortality rates of asthma have risen in most countries throughout the second half of the 20th century and then gone down with the implementation of ICS therapy and the introduction of modern guidelines to the management of asthma [1,48]. These advances underscore the critical nature of good pharmacologic care and guideline practice in lowering mortality related to asthma [21,43].

Nevertheless, even with the general decreases in mortality rates in the majority of high-income countries, asthma deaths still exist and are a significant issue in various parts of the world [5,8]. Recent analyses of the world indicate that healthcare access, medication access, and compliance with treatment regimens exist as factors that promote the persistent differences in asthma mortality in countries and populations [45,46]. In certain countries with low and middle income levels, the mortality rates of asthma have been relatively high because of the lack of healthcare facilities and access to appropriate medications against asthma [5,44]. These tendencies highlight the necessity of better global policies that would help to empower asthma care and provide more comprehensive early identification of high-risk patients, as well as to implement evidence-based management practices more consistently and universally to decrease the number of preventable asthma deaths [21,42].

Overview of the NRAD

Background and Objectives of NRAD

The NRAD was a historic study that was conducted in the United Kingdom to analyze the situation surrounding the occurrence of asthma-related deaths and determine the factors that contributed to the latter [21,41]. The review was commissioned by the Royal College of Physicians to advise the National Health Service (NHS) on the need to address the continuing cases of asthma deaths, even with the available effective therapies and established clinical recommendations [22]. It is commonly known that asthma is a disease that can be controlled to a great extent; thus, the continuation of asthma-related mortality posed a question about the possibility of missing certain aspects of clinical practice and healthcare provision [21,24]. 

The review aimed to find the trends in clinical practice leading to fatal asthma events and to determine how the recommendations offered by the currently existing BTS/SIGN guidelines were properly applied in everyday care [22,24]. Moreover, NRAD wanted to produce evidence-based suggestions that would help to better address asthma and increase the safety of patient care, as well as decrease preventable deaths related to asthma within a healthcare environment [41]. 

Methodology of NRAD Investigation

The NRAD inquiry was a secret national investigation that comprised a comprehensive review of asthma-related fatalities in the United Kingdom, which was retrospective in nature [21]. The analysis included deaths that were recorded between February 2012 and January 2013, where suspected asthma-related mortality cases were discovered based on the records of national death certification [22,41]. The primary care providers, hospitals, and other pertinent healthcare services were requested to provide medical records of the deceased patients so that an in-depth evaluation of clinical management before each of the deaths could be conducted [21].

The available clinical data were reviewed by an expert multidisciplinary panel, which included respiratory specialists, general practitioners, nurses, and other healthcare workers who reviewed the collected data to assess the quality of asthma care received before death [24,41]. Some of the aspects that were evaluated during the review process included severity of the disease, patterns of medication prescriptions, compliance with clinical guidelines, and healthcare utilization patterns preceding the fatal incident [21,22]. Special focus was on the identification of clinical and system-related factors that can be modified and lead to poor asthma outcomes or slowed asthma recognition [24,41]. In this thorough review process, NRAD was meant to offer a more in-depth insight into the asthma death situation and how clinical practice could be improved [21].

Key Findings from NRAD

This was shown by the results of NRAD, which revealed that a considerable percentage of premature asthma fatalities could be prevented, pointing out major shortcomings in routine asthma management and healthcare provision. The review of 195 asthma deaths revealed that most patients had already received suboptimal care before the fatal incident, including poor monitoring of disease control and lack of follow-ups by healthcare professionals [21,22,41]. NRAD also reported specific findings that underscore these deficiencies: 45% of patients did not seek or receive medical attention during the final fatal attack, 39% had been prescribed more than 12 SABA inhalers in the previous year, and 80% had no record of being provided with a personal asthma action plan [41]. These findings indicate that many deaths were associated with modifiable failures in routine asthma care [21,24].

Another challenge that was found in the NRAD report was the absence of regular asthma reviews, which restricted the possibilities of healthcare professionals to review the disease control and make changes accordingly. It was also found in the review that SABA was over-prescribed and overreliance on reliever therapy should prompt urgent review of asthma control, because it is commonly linked to poor asthma control and high risk of severe exacerbation and mortality [24,41]. On the other hand, a large portion of the patients who succumbed to asthma were reported to have been inadequately using ICS, which are crucial controller drugs in suppressing airway inflammation and severe attacks [22].

Moreover, NRAD also emphasized the inability to identify patients who were more likely to experience acute asthma manifestations, such as those with a history of hospitalizations, frequent attacks, or a lack of medication adherence [21,24]. Patient education, inhaler technique evaluation, and the distribution of written asthma action plans, which are one of the elements of effective self-management, were also found to be lacking in many cases [22]. A combination of these findings illustrated that much asthma mortality was within the environment of low compliance with the element of guideline-based asthma care, which emphasized the necessity to improve adherence to established clinical guidelines to prevent the emergence of preventable asthma mortalities [21].

Preventable factors identified in fatal and near-fatal asthma

The presence of preventable factors plays a critical role in the onset of fatal and near-life-threatening asthma attacks, and different studies have found that quite a large number of asthma-associated deaths occur in the company of modifiable risk factors that relate to patient behavior, clinical care, and failure of the healthcare system [21,41]. Research, such as the NRAD, notes that improper asthma management, failure to adhere to medical treatment, and poor recognition of exacerbation symptoms are typical antecedents of fatal asthma attacks [24,41]. The results show that the risk of experiencing severe exacerbation and asthma-specific mortality may be minimized by improving patient education, prescribed medication levels, and identifying poor control in asthma management [13,23]. Patient-related behaviors are an especially significant group of preventable risks among the other contributing factors in relation to fatal and near-fatal asthma outcomes [22].

Patient-Related Factors

Factors related to the patient lead to morbidity and mortality of asthma, especially when patients do not adhere to prescribed treatments or neglect to consult medical professionals when the disease becomes more problematic [24]. Poor compliance with ICS therapy is one of the most commonly reported risk factors related to fatal asthma, and it is the main concept used to regulate airway inflammation and avoid asthma exacerbations [1,23]. It has been demonstrated that patients not taking ICS drugs regularly face higher risks of having uncontrolled asthma and severe attacks that can result in respiratory arrest [4,41]. In the NRAD report, patients dying of asthma had been either under-prescribed ICS therapy or used it irregularly, which signifies a considerable traumatic fault in patient adherence, as well as in the monitoring of asthma [21].

The second patient-related risk factor, which is found to contribute to fatal and near-fatal asthma, is the overuse of SABA inhalers as commonly used reliever medications when acutely affected by asthma [22,24]. SABA inhalers, as a treatment modality, can attenuate deterioration of airway inflammation, but cannot address the pathophysiological mechanisms of asthma [1,23]. 

Overuse of SABA is thus considered one of the main indicators of out-of-control asthma and a risk indicator of severe exacerbations and asthma-related mortality [4,24].

The other important patient-related risk factor is smoking [8,43]. Exposure to tobacco smoke is also a contributor to airway inflammation, decreased corticosteroid therapy responsiveness, and faster lung dysfunction in asthmatic patients [4,43]. Research has shown that asthma patients who smoke have a higher likelihood of severe exacerbations, are more likely to get hospitalized, and have worse overall disease management than non-smokers [8,23]. Arguably, smoking cessation is therefore a critical element of asthma management and prevention of severe asthma-related complications [4,43].

Lack of awareness about the increasing symptoms of asthma also constitutes a significant cause of fatal and near-fatal asthma attacks [13]. This leads to patients putting off medical consultations until a late point when the exacerbation has intensified to an acute or life-threatening level [21]. As the NRAD study found, delayed presentation and an insufficient response to worsening symptoms were typical features of most fatal asthma cases [22]. These results highlight the importance of patient education and self-management, as well as individual asthma action plans, to raise awareness of asthma worsening warning signs and to respond to them [23].

Healthcare System-Related Factors

Factors associated with healthcare systems are a significant group of risk factors that lead to fatal and near-fatal asthma because failure to deliver healthcare and coordinate care can be a serious issue in determining the quality of asthma management and patient outcome [21,41]. Several studies, such as the NRAD, have shown that asthma mismanagement and subsequent risk of severe exacerbation and death can be caused by flaws in healthcare systems, including a lack of proper monitoring, care fragmentation, and follow-up [22].

The absence of regular asthma reviews was among the most common healthcare system failures in NRAD, as it is necessary to evaluate disease control and medication adherence and to revise treatment plans based on existing clinical guidelines. Consistent asthma reviews enable healthcare providers to track symptom progression, evaluate inhaler competence, and identify patients who may develop severe asthma [24,40]. Nevertheless, the NRAD report concluded that a significant proportion of asthma deaths did not receive routine asthma evaluations in primary care before the fatal incident, which means that they missed opportunities to intervene in time and manage the disease better [22].

The second issue that is very important in terms of healthcare systems and can lead to death with asthma is a lack of follow-up post-acute asthma attacks or hospitalizations [21,24]. Patients with the highest risk of recurrent attacks, as well as subsequent death, are those who develop severe asthma attacks requiring admission to the emergency department or hospital [13,23]. Clinical guidelines suggest that these patients should receive follow-up care promptly to review treatment, reinforce adherence to controller medications, and address modifiable risk factors [40]. Nevertheless, NRAD findings demonstrated that the proportion of patients dying as a result of asthma did not have proper post-exacerbation follow-up, which further predisposed them to the risk of uncontrolled disease and frequent severe attacks [21].

Lack of communication among healthcare providers is also a major issue in the healthcare system that can adversely affect asthma management. Asthma management can be challenging to achieve without collaboration among several medical professionals, including primary care providers, respiratory specialists, emergency department clinicians, and nurses [40]. Poor communication between such providers can lead to a situation in which key clinical data, including medication changes, exacerbation history, and risk factors for severe asthma, are not efficiently exchanged, resulting in fragmented care [22]. The NRAD report indicated that, in several cases, the lack of effective communication among healthcare providers led to the loss of opportunities to implement early intervention and enhance disease monitoring [41]. These results highlight the significance of multifaceted healthcare models, effective channels of communication, and multidisciplinary teams in reducing preventable asthma morbidity and mortality.

Medication-Related Factors

Other key aspects of avoidable contributors to fatal and near-fatal asthma are the medication-related factors, with improper medication practices and inappropriate prescribing being of critical importance in undermining asthma control and risking severe asthma exacerbation [21,41]. Pharmacologic treatment is a key factor in achieving good asthma control and involves the use of ICS as controller drugs and bronchodilators as reliever medications [50]. Nevertheless, clinical research and national studies like the NRAD show that prescribing, medication adherence, and inhaler technique are often deficient in patients with fatal outcomes of asthma [22,41]. According to the NRAD investigation, a significant percentage of asthma deaths involved patients who were prescribed excessive amounts of SABA inhalers during the previous year and were hence indicative of poorly controlled disease and improper utilization of reliever therapy [21,41]. These data underscore the need to monitor SABA use as an indicator of uncontrolled asthma and implement alterations in the treatment solutions [24,50].

Another medication-related risk factor, which could greatly diminish the outcome of asthma treatment and promote the inability to control the disease, is incorrect inhaler technique [51]. To achieve efficacy of medications to the lower airways, producing the therapeutic effect, medications must be delivered via the inhaler properly [4,51]. It has been determined, however, that a considerable percentage of asthmatic patients exhibit improper inhaler use, which may result in insufficient delivery of medication and poor treatment outcomes [51]. The NRAD report noted that the inhaler technique used was not a regular issue that was evaluated during medical check-ups, which is a missed chance to enhance medication effectiveness and patient outcomes [22,41]. The need to address the inhaler technique by educating the patient and reassessing it regularly is thus a significant part of overall asthma management and prevention of severe asthma-related complications [23,51].

Clinical Management Factors

The significance of clinical management factors in the outcomes of patients experiencing severe asthma attacks has been recognized, and the lack of acute clinical care has been noted to be of critical importance in fatal and near-fatal asthma attacks [41]. To provide effective clinical care, it is crucial to promptly identify asthma exacerbation symptoms, seek medical help, and properly evaluate patient-related risk factors to prevent disease progression and harmful complications [50]. Nevertheless, studies like the NRAD have shown that multiple clinical management errors often lead to asthma-related deaths, including delayed hospital presentation, acute treatment, and patient risk assessment [22]. These challenges need to be addressed to enhance the outcomes of asthma and preventable mortality due to severe exacerbations [21,23].

Delays in hospital presentation during severe asthma exacerbations are only one of several significant clinical management factors that can lead to rapid worsening and a high risk of respiratory failure [13,41]. Patients who develop worsening asthma symptoms may also delay seeking medical assistance due to an impaired understanding of symptom severity, unavailability of medical services, or insufficient assessment of the condition's severity [23,24]. Inquiry conducted by NRAD had established that the patients, in the instance of the majority of the fatal asthma attacks, only sought medical attention when exacerbation had advanced to a critical level, which limited the effectiveness of emergency treatment measures. Medical evaluation and prompt visit to a hospital therefore form part of successful management of asthma, particularly in individuals with severe or inadequately controlled asthma [4,50].

The other clinical management problem identified as a significant concern in asthma fatality cases is the absence of acute management during severe exacerbations, which can lead to delayed administration of bronchodilators, corticosteroids, or oxygen [21,24]. The immediate, aggressive treatment of acute severe asthma is necessary to reverse bronchoconstriction, reduce airway inflammation, and ensure adequate ventilation [50]. Inhaled bronchodilators, systemic corticosteroids, and continuous observation of the respiratory condition are clinical decisions that are recommended to be expedited in patients with severe asthma attacks [4]. Nevertheless, the NRAD report revealed that through failure to provide relevant treatment promptly, the absence of relevant diagnosis and management of acute asthma attacks within the healthcare environment was noted in several cases of asthma-related deaths [41].

Another significant factor that can contribute to the number of deaths related to asthma that can be avoided is the inability to correctly evaluate potential risk factors of severe outcomes. It is important to identify patients who are at risk of developing fatal asthma, such as those who experienced a prior near-fatal attack, frequent hospitalization, poor medication adherence, overuse of reliever medications, etc., to use specific preventive measures [13,50]. Nonetheless, the results of NRAD showed that healthcare providers have often overlooked such high-risk features during routine clinical examination, depriving them of the opportunity to intensify monitoring and intervention [22]. Careful clinical examination and frequent risk assessment are thus the main attributes of effective asthma management and play a significant role in preventing severe exacerbations and asthma-related mortality.

Role of BTS/SIGN guidelines in preventing asthma deaths

Key BTS/SIGN Recommendations

The BTS/SIGN guidelines provide evidence-based guidance on the diagnosis, treatment, and long-term management of asthma in both primary and secondary care. These recommendations will optimize asthma treatment outcomes by standardizing clinical practices and providing patients with effective pharmacological and non-pharmacological treatments based on the latest scientific evidence [33,34]. One of the central principles of the BTS/SIGN guidelines is the stepwise approach to the management of asthma, which implies adjusting the intensity of treatment based on symptom severity and asthma control [38]. This strategy emphasizes ICS as the main component of controller therapy for airway inflammation and for preventing severe exacerbations [37,50].

Along with pharmacologic therapy, the BTS/SIGN recommendations emphasize patient education, self-management support, and regular check-ups to achieve successful asthma control [33,36]. It is advised to regularly review asthma management, medication compliance, and inhaler skills to maximize treatment outcomes [34]. The guidelines further emphasize the need to identify patients who may be more susceptible to severe asthma attacks and to put preventive measures in place to avert the risk of life-threatening attacks [36]. These suggestions are similar to the recommendations of the NRAD, which noted that better adherence to guidelines could reduce the number of preventable asthma deaths and improve outcomes considerably [24,41].

Risk Assessment Strategies

Risk assessment is an essential element of asthma management that BTS/SIGN guidelines deem necessary, as once clinicians identify patients who are highly prone to severe exacerbations or asthma-related mortality, they can begin implementing specific preventive measures [36]. Risk assessment implies the consideration of multiple clinical and behavioral risk factors that could lead to poor asthma outcomes, such as a history of severe exacerbations, the frequent use of reliever inhalers, low adherence to medications, and a history of hospitalization associated with asthma [50]. The patients having a history of near-fatal asthma attacks or a history of intensive care hospitalization are deemed to be at an exceptionally high risk and must be closely monitored and receive more intense treatment plans [13].

It is also suggested by the BTS/SIGN guidelines to evaluate the modifiable risk factors that may cause uncontrolled asthma and severe exacerbations, including smoking, poor inhaler technique, and poor use of controller medications [34,37]. Frequent evaluation of these risk factors during clinical consultations would enable healthcare providers to personalize treatment regimens and apply relevant interventions to enhance disease management [33,36]. According to the NRAD report, the importance of systematically assessing risk factors was highlighted, as a significant number of patients who succumbed to asthma had risk factors that were not effectively addressed during routine medical care [21,41].

Use of Personalized Asthma Action Plans

The BTS/SIGN guidelines are categorical and state that personalized asthma action plans are a fundamental aspect of successful asthma self-management. Research has shown that patients undergoing personalized asthma action plans have higher levels of symptom control, reduced hospitalization, and a lower probability of severe exacerbations than those receiving no form of structured self-management guidance [33,52].

Asthma action plans often include information on recognizing the initial signs of an asthma exacerbation, changing reliever and controller medications, and identifying situations when urgent medical intervention is necessary [36]. As the NRAD investigation also pointed out, a written asthma action plan was not offered to many people who succumbed due to asthma [21]. Giving patients a tangible, individually tailored action plan is therefore an important strategy of allowing patients to take charge in managing their condition and, in turn, deal with worsening symptoms [34].

Monitoring and Follow-Up Strategies

According to the BTS/SIGN guidelines, to support stable disease control and avoid serious exacerbation, follow-up and constant monitoring are an inevitable component of asthma treatment [36,37]. Clinical follow-ups allow healthcare professionals to measure the level of symptom control, detect the effectiveness of the treatment, and detect possible barriers to successful asthma management, such as non-adherence to the medications or the incorrect use of an inhaler [33,34]. 

According to the BTS/SIGN guidelines, asthma sufferers should receive regular follow-up evaluation, especially after severe attacks or hospitalization, to ensure proper recovery and prevent new life-threatening situations [36]. The follow-up visits can incorporate the assessment of the frequency of symptoms, testing of lung functions, medication adherence, and the evaluation of the environmental or behavioral triggers that might lead to ineffective disease management [50]. The NRAD report also noted that a significant number of deaths among patients with asthma can be prevented by timely follow-ups after prior episodes of exacerbation, which is why the structured follow-up monitoring and regular interaction with patients are regarded as critical to preventable mortality in asthma [21,41].

Lessons learned from NRAD and BTS implementation

The results of the NRAD, along with the suggestions of the BTS/SIGN guidelines, have given significant information on how the management of asthma and avoidable asthma deaths can be addressed and improved. The preventable factors identified in NRAD are given in Table 1.

**Table 1:** Preventable factors identified in NRAD NRAD: National Review of Asthma Deaths; SABA: short-acting β₂-agonist

Category	Preventable factor	Description	References
Patient-related factors	Poor adherence to inhaled corticosteroids	Many patients who died from asthma were not regularly using inhaled corticosteroids, leading to uncontrolled airway inflammation and increased risk of severe exacerbations	[21,41]
	Excessive use of SABA	A large proportion of patients who died had been prescribed multiple SABA inhalers in the year before death, indicating poor asthma control	[24,41]
	Lack of personalized asthma action plans	Many patients had not been provided with written asthma action plans to guide self-management and early response to worsening symptoms	[21]
Clinical management factors	Failure to identify high-risk patients	Patients with previous hospitalizations or severe exacerbations were not always recognized as high-risk individuals requiring closer monitoring	[22]
	Inadequate assessment of asthma control	Routine evaluation of symptom control and disease severity was often lacking in primary care settings	[24,41]
Healthcare system factors	Lack of regular asthma reviews	Many patients had not undergone annual asthma reviews before their fatal exacerbation	[21,41]
	Inadequate follow-up after hospital admission	Patients discharged after severe exacerbations often did not receive timely follow-up care to reassess treatment plans	[24]
	Poor communication between healthcare providers	Fragmented care and limited information sharing between primary and secondary care providers contributed to gaps in patient management	[22,24]
Medication-related factors	Under-prescription of inhaled corticosteroids	Some patients were not prescribed adequate controller therapy despite evidence of poorly controlled asthma	[21,41]
	Lack of inhaler technique assessment	Incorrect inhaler technique was often not evaluated during clinical visits, reducing treatment effectiveness	[22]

These reports indicate that numerous asthma-related deaths happen within the framework of poor clinical care, patient deficits in disease monitoring, and patient deficits in self-management [22,24]. Analyzing these lapses in everyday asthma medicine, NRAD and BTS/SIGN guidelines highlight a number of essential lessons, which can be used to transform better clinical practice and medical care delivery to minimize the deaths caused by asthma [36]. BTS guideline recommendations for high-risk asthma are given in Table 2. 

**Table 2:** BTS guideline recommendations for high-risk asthma BTS: British Thoracic Society

Category	Guideline recommendation	Description	References
Risk assessment	Identification of high-risk patients	Clinicians should identify patients at increased risk of severe asthma exacerbations or death, including those with previous near-fatal asthma, frequent hospitalizations, or excessive reliever inhaler use	[21,37]
Pharmacologic management	Regular use of inhaled corticosteroids	Inhaled corticosteroids are recommended as the cornerstone of asthma treatment to control airway inflammation and prevent severe exacerbations	[37,50]
Pharmacologic management	Stepwise approach to therapy	Asthma treatment should follow a stepwise strategy, adjusting medication intensity based on symptom control and disease severity	[36,37]
Medication monitoring	Monitoring reliever inhaler use	Excessive use of short-acting β₂-agonists should trigger clinical review because it may indicate poorly controlled asthma and increased risk of severe attacks	[37]
Patient education	Provision of personalized asthma action plans	Patients should receive written asthma action plans to guide symptom monitoring, medication adjustments, and timely medical consultation during exacerbations	[52]
Clinical monitoring	Regular asthma reviews	Patients with asthma should undergo regular clinical reviews to assess symptom control, medication adherence, and inhaler technique	[36]
Post-exacerbation care	Follow-up after acute asthma exacerbations	Patients who experience severe exacerbations or hospital admissions should receive prompt follow-up to reassess treatment and prevent recurrence	[24]
Self-management support	Education on inhaler technique	Healthcare providers should regularly assess and correct inhaler technique to ensure effective medication delivery	[51]
Multidisciplinary care	Referral to specialist services when needed	Patients with severe or difficult-to-control asthma should be referred to respiratory specialists for advanced management	[33,36]

To provide a clearer synthesis of the review's central theme, Table 3 presents a cross-walk between NRAD-identified failures, corresponding BTS/SIGN recommendations, relevant updates in the 2024 BTS/NICE/SIGN guidance, and the outstanding gaps that continue to affect asthma care.

**Table 3:** Cross-walk between NRAD-identified failures and BTS/NICE/SIGN guideline recommendations NRAD: National Review of Asthma Deaths; BTS: British Thoracic Society; SIGN: Scottish Intercollegiate Guidelines Network; NICE: National Institute for Health and Clinical Excellence; SABA: short-acting β₂-agonist; ICS: inhaled corticosteroid

NRAD failure	BTS/SIGN recommendation	2024 update	Outstanding gap
Over-prescription of SABA	Regular review of symptom control and medication use	Greater emphasis on contemporary risk-based management and monitoring	Persistent overreliance on reliever therapy in routine care
Underuse of ICS	ICS as foundation controller therapy	Reinforced alignment with modern anti-inflammatory treatment principles	Adherence remains poor in real-world practice
Lack of regular review	Routine follow-up and monitoring	Continued emphasis on structured review	Inconsistent implementation across settings
Poor identification of high-risk patients	Risk stratification and closer monitoring	More explicit current framework for ongoing monitoring	High-risk patients may still be missed in primary care
Lack of written action plans	Personalized asthma action plans	Ongoing support for self-management and written guidance	Action plans remain underused
Delayed response to worsening symptoms	Patient education and early intervention advice	Continued focus on supported self-management	Delays in help-seeking still occur

Another necessary aspect that NRAD discloses is the necessity of regular asthma check-ups, as it allows medical workers to assess the level of disease control, to check the effectiveness of the treatment process, and to modify the management plan based on the needs of a patient. The periods between asthma follow-ups are the times when the recurrence of symptoms, compliance with medication, and inhaler technique can be verified, and they are essential in the management of asthma to the best [35]. The NRAD report also stated that many individuals who died due to asthma had not had routine clinical evaluations before their fatal exacerbation; therefore, opportunities for early intervention and more efficient monitoring of the illness were not offered [22]. The follow-up and structured asthma review is hence an important measure to prevent severe exacerbations and postpone asthma-related mortality [36].

The second valuable conclusion of NRAD is that better ways to identify high-risk patients with a higher risk of severe asthma outcomes are required [21,24]. The elderly patients who already had prior hospital admissions, experienced many exacerbations, and overused the reliever inhalers, as well as the patients with a history of near-fatal asthma attacks, constitute highly vulnerable groups, as they need careful monitoring and more aggressive management approaches [13,37]. The early identification of these risk predispositions helps healthcare professionals to establish specific interventions, such as optimal pharmacotherapy, increased follow-up care, and specialist care referral in cases of need [36]. In the NRAD study, it was found that risk factors were present in many patients who died of asthma and they were not properly taken into account during the usual clinical practice, which is why systematic risk assessment is an important issue in asthma management [21,41].

Patient education was also revealed as an important part of successful asthma management and the prevention of fatal asthma attacks. Patient education on the nature of asthma, the need to follow controller medications, and the timely identification of symptoms of worsening can help greatly to improve self-management and minimize chances of acute exacerbations [33,52]. According to the NRAD report, numerous patients did not receive written asthma action plans, and most of them did not have sufficient knowledge regarding their condition, which could have been one of the factors that led to delays in seeking medical attention during severe attacks [21,41]. The increase in patient education and the delivery of structured self-management support are thus crucial measures that should empower individuals to become proactive managers of their disease and avoid life-threatening exacerbations [34].

Another lesson that NRAD and BTS/SIGN guidelines teach to reduce asthma-related mortality is the improvement of prescribing practices. Pharmacologic management should be used to control airway inflammation and prevent severe asthma attacks by appropriately using ICS and closely monitoring inhaled reliever use [50]. The NRAD study found that a substantial number of patients dying of asthma were prescribed too many SABA inhalers and incorrectly prescribed controller therapy. The adoption of the therapeutic plans outlined in the guidelines and the frequent monitoring of prescribed medications can thus help identify patients with uncontrolled asthma and reduce the risk of adverse outcomes [36,37].

Strategies to reduce preventable asthma deaths

To curb unnecessary deaths caused by asthma, it is necessary to have comprehensive interventions that would cover patient-related, clinical management, and healthcare system shortcomings reported in research studies, including the NRAD and global asthma guidelines [21]. The prevention strategies must be aimed at early identification of high-risk patients, optimizing pharmacologic treatment, better patient education and self-management, and developing stronger healthcare systems to provide patients with timely and synchronized asthma care. All these strategies can help to manage asthma, lessen severe exacerbations, and eventually decrease mortality related to asthma [50].

Early identification of high-risk patients

One of the most important strategies that can be used to avoid fatal outcomes is the early detection of patients who are at risk of severe asthma exacerbations or mortality due to asthma. Clinical practice guidelines describe the need to identify risk factors, including the history of previous near-fatal asthma attacks, repeated hospitalization, overuse of reliever inhalers, and poor adherence to controller medications [50]. Such patients need more intensive surveillance, a tailored approach to treatment, and even referral to specialist respiratory professionals to thoroughly assess and address them. The NRAD report showed that a significant part of patients dying of asthma had risk factors that could be identified and could not be properly evaluated in the context of regular clinical care, which necessitates systematic risk assessment and initial intervention. Early detection of individuals who are at risk is crucial in minimizing the chances of excessive exacerbation and avoidable deaths from asthma [21,24,41].

Optimizing pharmacologic therapy

Optimization of pharmacologic therapy is central to preventing asthma mortality by reducing airway inflammation and minimizing the risk of severe exacerbations. ICS remain the cornerstone of asthma management due to their ability to suppress chronic airway inflammation and reduce bronchial hyperresponsiveness [37,50]. Current clinical guidelines recommend stepwise adjustment of controller therapy based on symptom control and exacerbation risk, with regular treatment review to ensure appropriate escalation or de-escalation [36].

Recent guideline updates, including the 2024 BTS/NICE/SIGN recommendations and the GINA strategy, have further shifted practice away from reliance on SABA-only reliever therapy. Instead, they support the use of anti-inflammatory reliever (AIR) therapy and maintenance-and-reliever therapy (MART) using low-dose ICS-formoterol, which provides both rapid symptom relief and concurrent anti-inflammatory treatment. This approach has been shown to reduce severe exacerbations and asthma-related hospitalizations compared with SABA-based regimens.

Despite these advances, excessive SABA use remains a marker of poor asthma control and increased risk of fatal or near-fatal attacks, reinforcing the importance of monitoring reliever use and ensuring timely escalation of controller therapy. Overall, adherence to contemporary guideline-based prescribing, including AIR/MART strategies where appropriate, is essential for optimizing asthma outcomes and preventing avoidable deaths [33].

Patient education and self-management

Self-management support and patient education are strategies that play a significant role in enhancing asthma control and reducing the risk of severe exacerbations [52]. Patient education on the nature of asthma, the necessity to take the controller medications regularly, and the need to recognize the signs of worsening conditions early can be one of the main strategies to improve patient adherence rates and allow them to seek medical help when symptoms aggravate. Personal written asthma action plans are particularly helpful when it comes to assisting patients to monitor their symptoms, manipulate medications, and seek medical care when necessary [34,52]. Providing evidence, it has been shown that patients undergoing structured self-management education with written action plans experience reduced hospitalizations and better overall asthma management compared to patients who do not receive such interventions. Enhancing patient education programs and marketing self-management measures are thus important elements in preventing lethal asthma outcomes [21,52].

Healthcare system improvements

Healthcare systems also need to be improved to focus on structural issues that cause death due to asthma that can be prevented. Improving healthcare infrastructure, providing access to critical asthma medications, and better coordination among primary and secondary care providers can improve asthma management and reduce care gaps [37]. The frequent examination of asthma and the systematic follow-up following exacerbations, in addition to enhanced communication between healthcare professionals, are required to monitor the worsening control of asthma and deliver timely interventions. Also, the interdisciplinary team of physicians, nurses, pharmacists, and respiratory specialists can support overall asthma care and enhance treatment adherence. Healthcare systems can also help reduce preventable morbidity and mortality from asthma at the global level by tackling systemic barriers to effective asthma management [21,24,33].

Limitations of the current evidence

Although more studies on asthma mortality and severe attacks are being conducted, the existing evidence is limited, as it can affect the impact of the findings on the number of asthma deaths that can be prevented [4,45]. The first is that epidemiological data for certain areas, especially those in low- and middle-income countries, might be less complete and the healthcare infrastructure and surveillance systems might not be as advanced [3,5]. As a result, the burden of asthma mortality in the world can be underestimated because of the lack of full reporting and access to diagnostic and monitoring services [4]. 

Underreporting of deaths caused by asthma also represents another limitation because asthma has not been properly noted as the principal cause of death on a medical record or a death certificate [48]. There can also be errors in the mortality statistics, as, in these instances, it is possible to attribute the deaths to complications, including respiratory failure, other than to the underlying exacerbation of asthma [5,48]. The absence of standardized systems for reporting and classifying countries may also lead to discrepancies in epidemiological data and restrict comparisons between studies [41].

Future directions

Future studies are to concentrate on creative methods of enhancing early recognition of aggravated asthma management and avert severe asthma attacks and death caused by asthma [50]. The new digital monitoring tools, such as mobile apps, smart inhalers, and remote monitors, can also be used to monitor symptoms and medication adherence and detect worsening of asthma management earlier [53,54]. Furthermore, biomarker-based therapy, with blood eosinophil counts and fractional exhaled nitric oxide (FeNO) as examples, can help manage asthma more effectively and achieve better outcomes in patients with severe or difficult-to-treat asthma [55,56].

The further development of artificial intelligence and predictive analytics can assist risk stratification by identifying patients at elevated risk of severe asthma exacerbations or death by analyzing large volumes of clinical data [57]. Furthermore, additional implementation research is warranted to ascertain the impediments to the practical application of the subsequent guidelines and to facilitate the integration of evidence-based recommendations into routine clinical settings [33,58]. These strategies can be further refined to promote improved asthma control and mitigate asthma-related mortality in the future.

Future efforts should also evaluate how far the BTS/NICE/SIGN 2024 recommendations address the gaps highlighted by NRAD and how effectively these updates are being implemented in routine clinical practice.

## Conclusions

Asthma-related deaths remain substantially preventable. The evidence reviewed here, together with NRAD findings, suggests that the most important modifiable contributors are underuse of controller therapy, overreliance on SABA, inadequate follow-up, poor self-management support, and failure to identify high-risk patients. Stronger implementation of guideline-based care and earlier risk recognition are likely to offer the greatest gains in reducing fatal and near-fatal asthma outcomes. Most fatal asthma outcomes are preventable with guideline-based care, early risk recognition, and stronger self-management support.
